# Germline Copy Number Variation and Ovarian Cancer Survival

**DOI:** 10.3389/fgene.2012.00142

**Published:** 2012-08-08

**Authors:** Brooke L. Fridley, Prabhakar Chalise, Ya-Yu Tsai, Zhifu Sun, Robert A. Vierkant, Melissa C. Larson, Julie M. Cunningham, Edwin S. Iversen, David Fenstermacher, Jill Barnholtz-Sloan, Yan Asmann, Harvey A. Risch, Joellen M. Schildkraut, Catherine M. Phelan, Rebecca Sutphen, Thomas A. Sellers, Ellen L. Goode

**Affiliations:** ^1^Department of Health Sciences Research, Mayo ClinicRochester, MN, USA; ^2^Biostatistics Department, University of Kansas Medical CenterKansas City, KS, USA; ^3^Department of Cancer Epidemiology, Moffitt Cancer CenterTampa, FL, USA; ^4^Department of Laboratory Medicine and Pathology, Mayo ClinicRochester, MN, USA; ^5^Department of Statistical Science, Duke UniversityDurham, NC, USA; ^6^Department of Biomedical Informatics, Moffitt Cancer CenterTampa, FL, USA; ^7^Case Comprehensive Cancer Center, Case School of MedicineCleveland, OH, USA; ^8^Department of Epidemiology and Public Health, Yale University School of MedicineNew Haven, CT, USA; ^9^Department of Community and Family Medicine, Duke University Medical CenterDurham, NC, USA; ^10^Pediatrics Epidemiology Center, College of Medicine, University of South FloridaTampa, FL, USA

**Keywords:** association testing, copy number variation, genotyping array, ovarian cancer, overall survival

## Abstract

Copy number variants (CNVs) have been implicated in many complex diseases. We examined whether inherited CNVs were associated with overall survival among women with invasive epithelial ovarian cancer. Germline DNA from 1,056 cases (494 deceased, average of 3.7 years follow-up) was interrogated with the Illumina 610 quad genome-wide array containing, after quality control exclusions, 581,903 single nucleotide polymorphisms (SNPs) and 17,917 CNV probes. Comprehensive analysis capitalized upon the strengths of three complementary approaches to CNV classification. First, to identify small CNVs, single markers were evaluated and, where associated with survival, consecutive markers were combined. Two chromosomal regions were associated with survival using this approach (14q31.3 rs2274736 *p* = 1.59 × 10^−6^, *p* = 0.001; 22q13.31 rs2285164 *p* = 4.01 × 10^−5^, *p* = 0.009), but were not significant after multiple testing correction. Second, to identify large CNVs, genome-wide segmentation was conducted to characterize chromosomal gains and losses, and association with survival was evaluated by segment. Four regions were associated with survival (1q21.3 loss *p* = 0.005, 5p14.1 loss *p* = 0.004, 9p23 loss *p* = 0.002, and 15q22.31 gain *p* = 0.002); however, again, after correcting for multiple testing, no regions were statistically significant, and none were in common with the single marker approach. Finally, to evaluate associations with general amounts of copy number changes across the genome, we estimated CNV burden based on genome-wide numbers of gains and losses; no associations with survival were observed (*p* > 0.40). Although CNVs that were not well-covered by the Illumina 610 quad array merit investigation, these data suggest no association between inherited CNVs and survival after ovarian cancer.

## Introduction

Epithelial ovarian cancer is the fifth leading cause of cancer death among women in the United States, accounting for 5% of cancer deaths (Jemal et al., 2011). Though rare, most patients are diagnosed with advanced disease due to the non-specific nature of early symptoms and lack of effective screening strategies. For the approximate 75% of women diagnosed with stage III or IV disease, the likelihood of long term disease-free survival ranges from 15 to 20% (Hoskins et al., 1992; McGuire et al., 2002; Barnholtz-Sloan et al., 2003). As women may inherently vary in their ability to eradicate disease or tolerate treatment, genetic association studies have sought to identify inherited variants related to overall survival. In 2010, the first ovarian cancer genome-wide association study (GWAS) to examine survival did not identify any replicated survival-associated single nucleotide polymorphisms (SNPs; Bolton et al., 2010).

Like SNPs, copy number variants (CNVs) occur commonly in the genome and have been implicated in risk of complex diseases including schizophrenia (Need et al., 2009), neuroblastoma (Diskin et al., 2009), and prostate cancer (International Schizophrenia Consortium, 2008; Liu et al., 2009). The size and frequency of the detected CNV (deletions) from these studies varied, with size of deletions ranging from intermediate (4 kb) to large (2 Mb). CNVs are a priori more likely to have larger phenotypic effects than SNPs (Cooper et al., 2008), and they have been shown to have adequate coverage on current SNP arrays, at least for large and intermediate size CNVs (CNVs > 5 kb; McCarroll, 2008). Therefore, to better understand inherited factors in ovarian cancer, we used the Illumina 610 quad array to characterize CNVs and evaluate associations with survival among over 1,000 ovarian cancer cases.

## Materials and Methods

### Study population

Participants were from three previously described studies of invasive epithelial ovarian cancer which enrolled cases from 2000 to 2008 from the upper Midwest [the Mayo Clinic Ovarian Cancer Study (MAY)], North Carolina [the North Carolina Ovarian Cancer Study (NCO)], and the Tampa Bay, FL, USA area (the Tampa Bay Ovarian Cancer Study, TBO; Permuth-Wey et al., 2011). Research protocols were approved by institutional review boards at each site, and all participants provided written informed consent. Cases were followed for vital status through 2009 using active contact, medical record review, and linkage to the National Death Index.

### Genotyping and quality control

DNA extracted from blood were genotyped at the Mayo Clinic Genotyping Shared Resource Facility (Rochester, MN, USA) using the Illumina Infinium 610 quad array as described previously (Permuth-Wey et al., 2011). Genotyping was attempted for 4,169 samples including ineligible participants (population controls, case without follow-up data, laboratory controls). Samples with call rate <95%, ambiguous gender, unresolved identical genotypes, self-reported non-Caucasian race, or less than 80% European ancestry as predicted by structure (Pritchard et al., 2000) analysis were excluded. This resulted in a sample size of 3,715, including 1,056 for the current analysis. Markers on the X chromosome or with call rate <80% were excluded, leaving 599,820 markers (581,903 SNPs, 17,917 CNV probes) for CNV analysis.

### Normalization of intensity data

Normalization of allelic intensities was completed similar to the approach used by Barnes et al. (2008). Let *A_ij_* and *B_ij_* represent the intensities of the two alleles for marker j measured on subject i. First, systematic differences between the *A* and *B* intensities were corrected using data for subjects with heterozygous genotype calls, with correction factor for marker *j* equal to ${\text{ψ}}_{j}=\left(\frac{1}{K}\right)\sum_{i=1}^{K}\left(\frac{A_{ij}}{B_{ij}}\right)$, with *K* representing the number of subjects with heterozygous genotype calls for marker *j*. This correction factor was then used to compute the total intensity for marker *j* measured on subject *i*, $I_{ij}=\log_{2}(A_{ij}+{\text{ψ}}_{i}B_{ij})$.

Next, a two-step normalization procedure was completed to remove plate and other experimental artifacts using quantile normalization to produce similar intensity distributions for each subject, followed by a median normalization for each marker by plate. Using normalized intensity values, $Z_{ij}=I_{ij}-{\bar{I}}_{i}$ was computed for each marker *j* measured on subject *i*, where ${\bar{I}}_{i}$ is the mean intensity for subject *i* computed over all the markers (mean intensity for the subject).

### Parameterization of CNVs

In order to comprehensively address the genetic architecture of CNV association with ovarian cancer survival, CNVs were characterized using three complementary approaches, as simulation showed different approaches to have maximal statistical power under varied genetic models (Breheny et al., 2012).

First, to identify associations with small, common CNVs, normalized intensities at individual markers (*Z*) were evaluated and, where associated with survival (association methods described below), results across neighboring genetic loci were combined (Ionita-Laza et al., 2008). To combine results at consecutive markers, we performed fused lasso regression of −log_10_ (*p*-values; Tibshirani et al., 2005), using the R package *cghFLasso*.^1^ In regions of interest [smooth −log_10_ (*p*-value) >2], chromosomal segmentation was completed using Partek^®^ Discovery Suite™ (version 6.3) to more precisely define the associated CNV boundaries (parameter settings: minimum of five markers, *p*-value threshold = 0.001, signal to noise ratio = 0.3, region below = −0.3, region above = 0.15).

Second, to identify associations with large, rare CNVs, genome-wide segmentation was conducted to define chromosomal gains and losses, and association with survival was evaluated by segment. The number of copies present at each chromosomal segment for each individual was estimated based on normalized intensities (*Z*; i.e., CNVs were “called”) using circular binary segmentation (CBS) methods of the R package *DNAcopy* with a three marker minimum^2^ (Venkatraman and Olshen, 2007). The R package *cghMCR* was used to determine common CNV regions and combine adjacent regions^3^ (Aguirre et al., 2004; lower threshold = 3rd percentile, upper threshold = 97th percentile, required recurrence = five). At every segment for which copy number was variable, association testing was conducted (association methods described below).

Finally, because markers or regions may not individually predict ovarian cancer survival, we also evaluated the general amount of copy number changes across the genome (i.e., CNV burden; Kathiresan et al., 2009). Genome-wide CNV burden for each participant was defined based on CBS of normalized intensities (*Z*) in three ways: the total number of gains or losses, the total number of gains, and the total number of losses. Association testing was then conducted for these three values (association methods described below). However, a limitation of this burden analysis approach is that the sizes of the CNVs are not taken into account during the analysis, as estimation of CNV breakpoints (and sizes) based GWAS data is difficult.

### Association testing

Association with overall survival was completed using Cox proportional hazards regression, accounting for left truncation, with estimation of hazard ratios (HRs) and 95% confidence intervals (CIs; Therneau and Grambsch, 2000). Time at risk was defined as date of diagnosis to death with censoring at last follow-up. As described above, the CNV variable of interest was either the normalized intensity at each marker (*Z*), the number of copies of certain chromosomal sections, or one of three measures of CNV burden. To control for population stratification, the first principal component from eigen-analysis of non-Hispanic white participants (Price et al., 2006; Permuth-Wey et al., 2011) was included as a covariate along with study site and age at diagnosis.

## Results

With an average 3.7 years of follow-up, 494 recurrences or deaths were observed among 1,056 successfully genotyped invasive epithelial ovarian cancer cases with follow-up for disease outcome. As shown in Table 1, 62% of cases were of serous histology, 71% were diagnosed at advanced stage, and the majority of subjects were enrolled within 4 months of diagnosis.

**Table 1:** **Clinical characteristics of invasive epithelial ovarian cancer cases**.

	MAY (*N* = 352)	NCO (*N* = 352492)	TBO (*N* = 352212)	Total (*N* = 3521,056)
	
	*N* (%)	*N* (%)	*N* (%)	*N* (%)
Deceased	171 (49)	229 (47)	94 (44)	494 (47)
**STAGE (FIGO)**				
I	67 (19)	125 (25)	36 (17)	228 (22)
II	26 (7)	36 (7)	16 (8)	78 (7)
III	199 (57)	314 (64)	139 (66)	652 (62)
IV	56 (16)	15 (3)	21 (10)	92 (9)
Missing	4 (1)	2 (0)	0 (0)	6 (1)
**GRADE**				
I	13 (4)	65 (13)	15 (7)	93 (9)
II	45 (13)	137 (28)	38 (18)	220 (21)
III	288 (82)	278 (57)	159 (75)	725 (69)
Missing	6 (2)	12 (2)	0 (0)	18 (2)
**HISTOLOGY**				
Serous	221 (63)	293 (60)	138 (65)	652 (62)
Endometrioid	71 (20)	81 (16)	27 (13)	179 (17)
Clear Cell	24 (7)	58 (12)	11 (5)	93 (9)
Mucinous	11 (3)	20 (4)	12 (6)	43 (4)
Mixed/Other	25 (7)	40 (8)	19 (9)	84 (8)
Missing	0 (0)	0 (0)	5 (2)	5 (0)

	**Mean (range)**	**Mean (range)**	**Mean (range)**	**Mean (range)**

Age at diagnosis	61.4 (28–91)	57.1 (22–74)	61.5 (26–93)	59.4 (22–93)
Days from diagnosis to enrollment	35.5 (0–835)	102.7 (5–1,178)	99.3 (0–947)	79.6 (0–1,178)
Years from diagnosis to last follow-up	3.3 (0.02–9.5)	4.6 (0.5–10.0)	2.5 (0.02–8.3)	3.7 (0.02–10.0)

**Figure 1:**
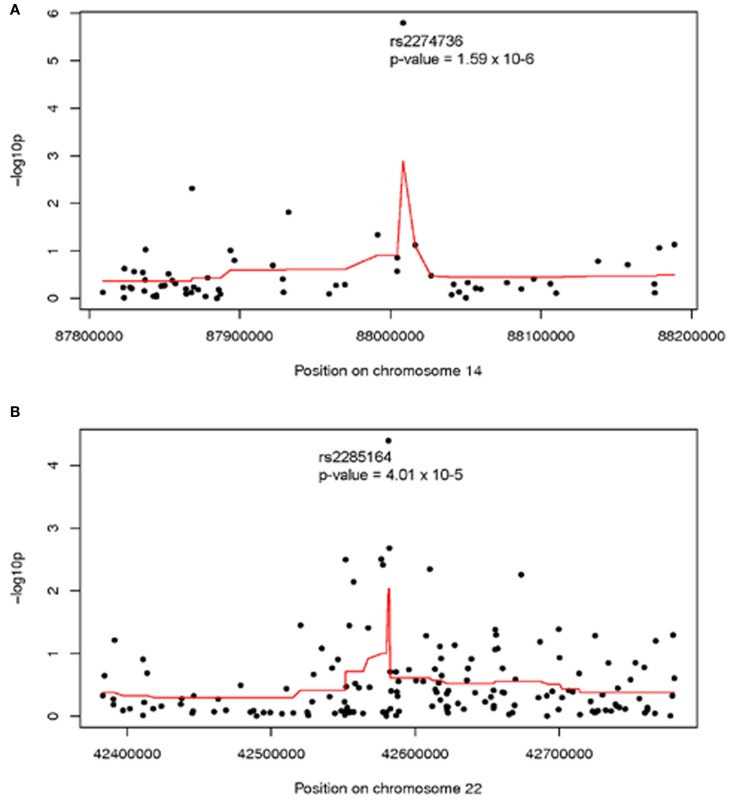
**Association between ovarian cancer survival and normalized intensities at individual markers (black dots) and smoothed regional association (red line) in (A) 14q31 and (B) 22q13; Genomic Build 36**. Analysis adjusted for study site, age at diagnosis, and first two population structure principal components.

Analysis of individual normalized marker intensities followed by combination of results across multiple consecutive markers is the most powerful approach for the detection of associations with small, common CNVs. Two regions showed suggestive association with ovarian cancer survival at multiple markers [smoothed −log_10_ (*p*) >2]. On 14q31.3 (379 kb, 63 markers) a smoothed *p* = 0.001 was observed suggesting a modest regional association with survival. As shown in Figure 1A, normalized intensities at rs2274736, a non-synonymous SNP in *PTPN21*, alone appeared to be driving the regional association. In fact, this SNP was the most significant single marker in genome-wide analysis (*p* = 1.6 × 10^−6^); note, however that it did not reach traditional genome-wide significance. Genomic segmentation in 14q31.3 was then done to identify specific gains or loss among study participants; however, only two samples were detected with gains and 18 samples with losses. Due to the lack of called CNVs in this region, further analysis was not carried out in 14q31.3.

**Figure 2:**
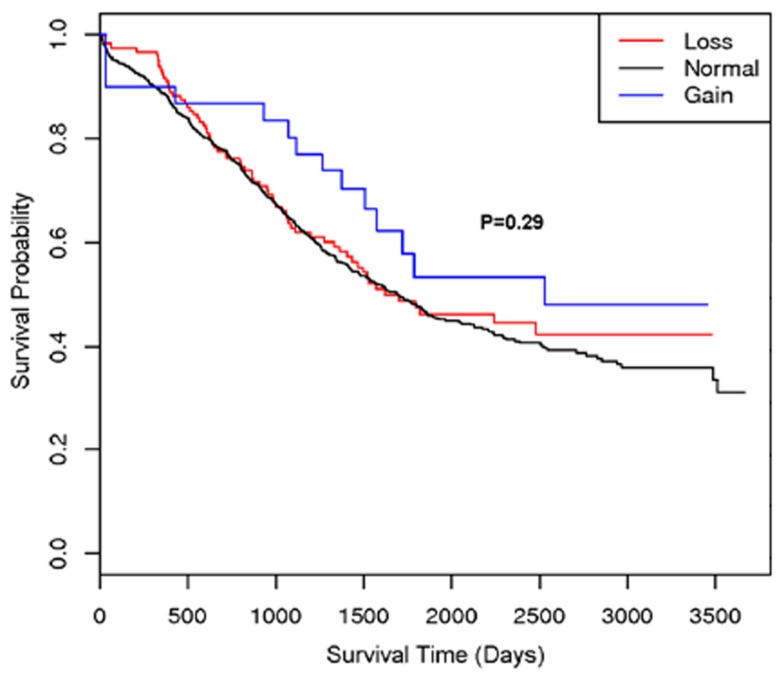
**Kaplan–Meier plot of the 22q13 CNV association with ovarian cancer survival**. The different lines represent the survival curve for subjects with a “loss,” “gain,” or “normal” for the genomic segment.

A second region with suggestive association with cancer survival at multiple markers was on 22q13.31 centered at rs2285164 (smoothed *p* = 0.009, 397 kb, 160 markers; Figure 1B). Genomic segmentation of 22q13 was then computed to identify specific gains or loss among study participants (Inc, 2008), where 35 showed gain and 154 showed loss. Association testing of gain (*N* = 35), normal (*N* = 867), or loss (*N* = 154) with survival did not reveal association (*p* = 0.29 for two degrees of freedom test; treating CNV as a categorical variable; *p* = 0.67 for one degree of freedom trend test treating CVN as a continuous variable). Thus, even though a signal was observed for association from the single marker analysis on 22q13, CNV calling, and subsequent analysis of this region showed no association between CNV and overall survival (Figure 2).

An inverse approach analyzing pre-defined regions of CNV change is most powerful for the detection of associations with large, rare CNVs. Genome-wide CBS identified 564 regions with variable copy number among the study population, including 78 regions with gain and 486 regions with loss (available upon request). Association testing of these regions revealed 14 regions with *p*-values <0.05, including one region of gain and 13 regions of loss (Table 2). Results at the most statistically significant regions (*p* = 0.002) suggested that loss of a region on 9p23 was associated with poorer survival (HR 1.44, 95% CI 1.14–1.81) as was gain of a region on 15q22.31 (HR 1.34, 95% CI 1.11–1.61). However, no region was statistically significant after correction for multiple testing using a Bonferroni procedure (564 tests).

**Table 2:** **Association between CNV region and ovarian cancer survival (*p* < 0.05)**.

Chr	Location (bp)	*N* Gain	*N* Normal	*N* Loss	HR (95% CI)	*p*-Value
1p31.1	72,538,673–72,549,855	0	992	64	1.52 (1.08–2.15)	0.017
1q21.3	151,026,302–151,033,105	0	947	109	1.46 (1.12–1.89)	0.005
4p15.31	19,130,834–19,131,054	0	994	62	1.48 (1.03–2.11)	0.032
5p14.1	27,462,485–27,462,654	0	975	81	1.54 (1.15–2.07)	0.004
6p21.33	30,017,499–30,017,538	0	1001	55	1.44 (1.01–2.06)	0.043
6p21.32	32,756,221–32,758,787	0	996	60	1.44 (1.01–2.05)	0.045
6q24.1	141,045,394–141,083,022	0	969	87	1.45 (1.07–1.94)	0.015
7q11.21	65,415,344–65,417,964	0	948	108	1.35 (1.03–1.76)	0.031
7q31.1	109,237,359–109,238,466	0	940	116	1.34 (1.04–1.74)	0.026
8q13.3	70,795,078–70,795,945	0	893	163	0.75 (0.57–0.97)	0.031
9p23	11,398,647–11,398,865	0	906	150	1.44 (1.14–1.81)	0.002
12q12	38,668,966–38,671,005	0	969	87	1.43 (1.07–1.91)	0.015
12q23.1	95,745,302–95,745,411	0	963	93	0.62 (0.43–0.90)	0.013
15q22.31	63,701,811–63,710,800	339	717	0	1.34 (1.11–1.61)	0.002

*HR, hazard ratio; CI, confidence interval adjusted for study site, age at diagnosis, and first two population structure principal components*.

*The start and end point based on the minimum common region determined from R package *cghMCR* (Aguirre et al., 2004), genomic build 36; normal number of copies is the reference group*.

Because the overall amount of variation from normal copy number across the genome (CNV burden) may contribute to disease, CNV burden for each case was estimated as summarized in Table 3. There was no association between survival and number of gains (*p* = 0.42), number of losses (*p* = 0.94), or total number of gains and losses (*p* = 0.84).

**Table 3:** **Mean genome-wide CNV burden by vital status and association with overall survival**.

	Alive (*N* = 562)	Deceased (*N* = 494)	HR (95% CI)	*p*-Value
			
	Mean (SD)	Mean (SD)		
*N* Losses	46.96 (22.47)	47.98 (20.76)	0.9998 (0.9958–1.0039)	0.94
*N* Gains	11.85 (6.75)	12.51 (6.85)	1.0051 (0.9926–1.0179)	0.42
Total *N* Gains and Loses	58.80 (23.35)	60.49 (21.44)	1.0000 (0.9997–1.0002)	0.84

**p*-Value, hazard ratio (HR), and confidence intervals (CI) from hazards regression adjusted for study site, age at diagnosis, and first two population structure principal components*.

## Discussion

Single nucleotide polymorphisms GWASs have yielded great insights into the etiology several complex diseases including ovarian cancer^4^ (Song et al., 2009; Bolton et al., 2010). However, germline variation related to disease outcome has been more elusive, with the possible exception of acute lymphocytic leukemia.^5^ With much unexplained variation in ovarian cancer outcome, investigation of additional inherited factors remains warranted. Here, in the first germline ovarian cancer CNV analysis, we have harnessed CNV data gleaned from dense genome-wide SNP genotyping in order to address the hypothesis that copy number variation is associated with survival. With over 1,000 cases (almost 500 deaths) representing three study populations, results were largely null suggesting no strong associations between small CNVs, large CNVs, or general CNV burden and ovarian cancer outcome.

There are numerous strengths to this study. First, the Illumina Infinium 610 quad array included markers in known regions according to the Toronto Database of Genomic Variation with approximately 38 markers per CNV region, markers in the “unSNPable” genome, markers in novel CNV regions, and intensity-only probes resulting in an average (median) coverage of 93% (100%) for white non-Hispanic populations.^6^ Second, our analysis leveraged a large sample size providing power to detect a HR of 1.75 for a CNV with frequency of 20% at a significance level of 10^−5^ (or HR = 2.00 at *p* < 10^−7^). Third, based on the available SNP data, assessment, and correction for possible population stratification to eliminate spurious associations due to confounding was performed. Fourth, extensive normalization of the intensity data from the SNP array was completed in order to produce reliable CNV calls. Finally, three complementary approaches for assessment and testing association between CNVs and ovarian cancer survival were utilized.

However, we cannot rule out more modest associations (HR < 2.0) between CNVs and ovarian cancer survival, and we cannot rule out associations with very small CNVs (<10 kb) which were not covered in Infinium 610 quad array. Despite this study being the largest of its kind to determine germline CNVs associated survival, the sample size was still relatively small and restricted to subjects of European ancestry. Finally, it is possible that CNVs exhibit survival effects only in the context of certain chemotherapeutic regimens or only among certain subtypes; these analyses are important and may reveal associations masked in the current analysis.

In conclusion, this study did not detect any CNVs associated with ovarian cancer survival using the data from the Infinium 610 quad array in three populations. Future research may reveal whether germline CNVs play a role in ovarian cancer etiology or in outcome within certain clinical contexts.

## Conflict of Interest Statement

The authors declare that the research was conducted in the absence of any commercial or financial relationships that could be construed as a potential conflict of interest.

## Footnotes

^1^http://cran.r-project.org/web/packages/cghFLasso/

^2^http://www.bioconductor.org/packages/release/bioc/html/DNAcopy.html

^3^http://www.bioconductor.org/packages/release/bioc/html/cghMCR.html

^4^http://www.nature.com/ng/journal/v42/n10/full/ng.668.html

^5^http://www.nature.com/ng/journal/v43/n3/full/ng.763.html

^6^www.illumina.com
